# Somatic alterations of *TP53* and *MDM2* associated with response to enfortumab vedotin in patients with advanced urothelial cancer

**DOI:** 10.3389/fonc.2023.1161089

**Published:** 2023-04-05

**Authors:** Tanya Jindal, Xiaolin Zhu, Rohit Bose, Vipul Kumar, Edward Maldonado, Prianka Deshmukh, Chase Shipp, Stephanie Feng, Michelle S. Johnson, Austin Angelidakis, Daniel Kwon, Hala T. Borno, Ivan de Kouchkovsky, Arpita Desai, Rahul Aggarwal, Lawrence Fong, Eric J. Small, Anthony Wong, Sima Porten, Jonathan Chou, Terence Friedlander, Vadim S. Koshkin

**Affiliations:** Helen Diller Family Cancer Center, University of California San Francisco, San Francisco, CA, United States

**Keywords:** urothelial carcinoma, antibody drug conjugate (ADC), enfortumab vedotin, genetic markers, next generating sequencing

## Abstract

**Background:**

Enfortumab vedotin (EV) is an antibody-drug conjugate approved for patients with treatment-refractory advanced urothelial carcinoma (aUC), however data on biomarkers of response is lacking.

**Methods:**

We retrospectively identified all aUC patients at our institution who received EV monotherapy and had next-generation sequencing (NGS) data available. Patients were considered responders if they had a complete response or partial response on restaging scans during treatment. Observed response rate (ORR) was evaluated by local investigator and compared between responders and non-responders using Chi-squared test. A univariable analysis was conducted using the Cox proportional hazard test to assess for associations between baseline characteristics and most common somatic alterations (in ≥10% of patients) with patient survival outcomes [progression-free survival (PFS) and overall survival (OS)]. Somatic alterations were then individually evaluated in separate multivariate models while accounting for patient and clinical characteristics using Cox regression models.

**Results:**

Among 29 patients treated with EV monotherapy, 27 had available NGS data. Median age was 70, 24 (83%) were men, 19 (62%) were Caucasian, 15 (52%) had pure urothelial histology and 22 (76%) had primary tumor in the bladder. ORR was 41%, and PFS and OS for the overall cohort were 5.1 months and 10.2 months. Responders were enriched among patients with *TP53, KDM6A* and *MDM2* alterations. Patients with these alterations, as well as those with composite *TP53/MDM2* alterations (alterations in either *TP53* or *MDM2*), also had increased ORR with EV treatment compared to patients without these alterations. In the univariable analysis, baseline albumin level ≥ 3.0g/dL and presence of composite *TP53/MDM2* alterations were associated with a prolonged OS. Baseline ECOG 0/1, *TP53* alterations and *TP53/MDM2* alterations were associated with a prolonged PFS. In the multivariable analysis, *TP53* and *TP53/MDM2* alterations were genomic markers predictive of improved PFS after accounting for the relevant clinical characteristics.

**Conclusion:**

In this single-center retrospective analysis of aUC patients treated with EV, presence of *TP53* or *MDM2* somatic alterations, lower ECOG PS scores (ECOG 0 or 1) and higher albumin levels (≥3 g/dL) were associated with improved outcomes with EV treatment. Prospective and external validation of these findings in larger cohorts is warranted.

## Introduction

Metastatic urothelial carcinoma (UC) is an aggressive and generally incurable malignancy. Within the past decade multiple new treatment options have been added to an armamentarium that until 2016 included only cytotoxic chemotherapy for patients with advanced UC. Patients with locally advanced or metastatic urothelial cancer undergoing treatment today have multiple other therapeutic options available, ranging from immune checkpoint inhibitors (ICIs) - pembrolizumab, nivolumab and avelumab, to antibody drug conjugates (ADCs) – enfortumab vedotin (EV) and sacituzumab govitecan (SG), and to targeted therapies such as erdafitinib for a subset of molecularly selected patients (1–10).

Enfortumab vedotin, one of the recently approved ADCs, is being increasingly used for patients whose cancers are refractory to platinum-based chemotherapy and ICIs, or patients who have ICI-refractory cancer and are not eligible for cisplatin-based chemotherapy. EV is composed of a fully humanized monoclonal antibody conjugated to monomethyl auristatin E (MMAE) *via* a cleavable linker. EV targets Nectin-4, a transmembrane protein that is highly expressed on the surface of urothelial cancer cells. It binds to Nectin-4 expressing cells, causing internalization of the drug followed by proteolytic cleavage of MMAE, which then leads to the disruption of microtubule networks and causes apoptotic cell death (9, 11). Initial promising data for EV was generated in the EV-101 phase I study, and in late 2019 the drug received accelerated FDA approval for treatment of patients with aUC after progression on prior platinum-based and ICI regimen based on the results of the phase 2 EV-201 trial (11, 12). The full FDA approval was later granted in 2021 following completion of the randomized phase 3 EV-301 trial (9). EV is now also being introduced in earlier treatment settings, with promising data emerging from the EV-103 study (13–19). The biomarker data reported in these trials has been quite limited and genetic and molecular biomarkers of response to EV are currently lacking.

As the therapeutic landscape for patients with aUC is expanding, additional guidance is needed to help define the optimal sequence of treatments that are now available for this patient population. Understanding which patients are most likely to benefit from EV treatment and which patients can be prioritized for other therapies is therefore of paramount importance.

In recent years, genomic profiling is increasingly being utilized in the management of patients with advanced solid tumors, including aUC (20–22). Our hypothesis was that using tumor genomic profiling data from available next generation sequencing (NGS) platforms in combination with clinical and laboratory data would identify alterations predictive of EV treatment outcomes.

## Methods

### Patient and data collection

Patients with aUC treated with enfortumab vedotin (EV) at the University of California, San Francisco from January 2020 through August 2022 were included in this analysis. Approval for this retrospective study was obtained from the institutional review board (IRB). The primary objective of this study was to investigate potential biomarkers of response to EV in patients with aUC.

Patients included in this analysis were required to have histologically confirmed diagnosis of UC, to have received at least one dose of EV, and to have had adequate treatment and response data in the electronic medical record (EMR). For response assessment, a patient was required to have at least one restaging scan after therapy initiation or have clear evidence of disease progression after therapy initiation as assessed by the treating physician. Most patients completed the initial post-treatment scan after 2 cycles of EV, though the precise timing of the scan was not mandated for study entry. Baseline clinicopathologic, laboratory, as well as tumor genomic profiling results for each patient were abstracted from the EMR in compliance with the IRB guidelines. Tumor genomic profiling was performed using Clinical Laboratory Improvement Amendments (CLIA)-certified next generation sequencing assays (FoundationOne or the institutional UCSF 500 Cancer Gene Panel Test platform).

Patients were classified as responders if they had a complete response (CR) or a partial response (PR) on restaging scans at any point during treatment, while the remaining patients with stable disease (SD) and/or progressive disease (PD) were classified as non-responders. The response assessment of observed response rate (ORR), defined as CR or PR was determined based on the judgement of the investigator who assessed the clinical notes and results from conventional radiology imaging adhering as closely as possible to the RECIST v1.1 criteria. No central radiology review was conducted, and scans were not collected at regular intervals for all patients. Given this limitation, progression-free survival (PFS) was defined as the time from treatment start to progression or death, and patients alive without disease progression were censored at the date of last follow-up. Overall survival (OS) was defined as the time from treatment start until death, and those alive were censored at the date of last follow-up.

### Statistical analyses

Summary statistics were used to assess the baseline patient clinical and treatment characteristics, genomic alterations identified by genomic profiling when available, as well as ORR. Univariable analysis was performed using cox proportional hazard test to assess for correlation between clinical outcomes (OS and PFS) and 1) relevant baseline demographic and clinical patient characteristics as well as laboratory values and 2) somatic alterations present in ≥10% patients.

Relevant baseline demographic and clinical patient characteristics included in the univariate analysis were: age, race, histology, primary tumor location, Eastern Cooperative Oncology Group (ECOG) performance status (PS) score, presence of visceral metastases, smoking status, and body mass index (BMI). The laboratory values assessed were: albumin, hemoglobin, neutrophil to lymphocyte ratio (NLR), and estimated glomerular filtration rate (eGFR). Two other prognostic factors which are a combination of baseline clinical and laboratory variables were also assessed: i) Bellmunt criteria (Hgb <10 g/dL, ECOG PS > 0, presence of liver metastases) (23) and ii) Frontline ICI risk score (NLR > 5, Albumin < 3.5 g/dL, ECOG PS ≥ 2, and presence of liver metastases) (24). These characteristics were chosen based on their clinical relevance for the patient population being studied and prior data associating some of these factors to clinical outcomes with other therapies in aUC patients.

Outcomes were also assessed based on the presence or absence of the most common somatic alterations (restricted to those present in ≥10% patients). Chi-squared test was used to assess differences in ORR among patients with and without the somatic alterations, and the Kaplan Meier Method was used to generate the OS and PFS curves comparing patients with and without a given somatic alteration.

To assess for independent effect of specific clinical and genomic variables on treatment outcomes with EV, multivariate cox proportional hazard models were then used to measure time-to-event outcomes (PFS and OS). Six pre-specified variables were selected for the multivariable analyses based on their clinical relevance and results of the univariate analyses: age, race, histology, presence of visceral metastases, ECOG score, and albumin level. OS and PFS were assessed for each somatic genomic alteration individually in a multivariate model while accounting for the above clinical variables. All statistical analyses were performed by RStudio (version 2022.07.0 Build 548). Statistical significance was set at a p<0.05 using two-sided tests. Adjustment for multiple testing was not performed.

## Results

### Baseline patient characteristics and treatment outcomes

Twenty-nine patients with aUC treated with EV monotherapy were identified. Of these 29 patients, 12 (41%) patients were classified as responders, including 4 (14%) with CR and 8 (28%) with PR. An additional 9 patients (31%) had SD as their best response, with an observed disease control rate (DCR) of 72%. Baseline demographic and clinical characteristics of the overall cohort and for responders and non-responders are summarized in **Table 1**. Fourteen (48%) patients in this cohort have received ≥ 2 prior lines of therapy before EV. Twenty-three (79%) patients were previously treated with platinum-based chemotherapy and 10 (34%) with immunotherapy.

**Table 1:** Patient demographics and clinical characteristics at start of EV therapy.

	Overall Cohort (N= 29)	Responders (N=12)	Non-Responders (N=17)
Median age (years)	70	74	69
**Gender – n (%)**			
Male	24 (83%)	10 (83%)	14 (82%)
Female	5 (17%)	2 (17%)	3 (18%)
**Race/Ethnicity– n (%)**			
Asian	8 (28%)	2 (17%)	6 (35%)
Black or African American	2 (7%)	0 (0%)	2 (12%)
Hispanic or Latino	1 (3%)	1 (8%)	0 (0%)
White	18 (62%)	9 (75%)	9 (53%)
**Primary tumor location – n (%)**			
Bladder	22 (76%)	9 (75%)	13 (76%)
Upper Tract	5 (17%)	2 (17%)	3 (18%)
Urethra	2 (7%)	1 (8%)	1 (6%)
**Histology – n (%)**			
Pure Urothelial	13 (45%)	7 (58%)	6 (35%)
Variant Component	14 (48%)	5 (42%)	9 (53%)
Pure Variant	2 (7%)	0 (0%)	2 (12%)
**Smoking history (present or former) – n (%)**	20 (69%)	8 (67%)	12 (71%)
**Median BMI (kg/m²)**	23.97	24.08	24.43
**Visceral metastases – n (%)**	21 (72%)	8 (67%)	13 (76%)
**Bone metastases – n (%)**	12 (41%)	4 (33%)	8 (47%)
**Liver metastases – n (%)**	9 (31%)	3 (25%)	6 (35%)
**ECOG – n (%)**			
0 or 1	19 (66%)	9 (75%)	10 (59%)
≥ 2	9 (31%)	2 (17%)	7 (41%)
Unknown	1 (3%)	1 (8%)	0 (0%)
**Hemoglobin – n (%)**			
< 10 g/dL	6 (21%)	2 (17%)	4 (24%)
≥ 10 d/dL	20 (69%)	9 (75%)	11 (65%)
Unknown	3 (10%)	1 (8%)	2 (12%)
**Albumin – n (%)**			
< 3 g/dL	6 (21%)	2 (17%)	4 (24%)
≥ 3 g/dL	20 (69%)	9 (75%)	11 (65%)
Unknown	3 (10%)	1 (8%)	2 (12%)
**eGFR – n (%)**			
< 60 mL/min/1.73m2	17 (59%)	8 (67%)	9 (53%)
≥ 60 mL/min/1.73m2	9 (31%)	3 (25%)	6 (35%)
Unknown	3 (10%)	1 (8%)	2 (12%)
**NLR – n (%)**			
< 5	17 (59%)	5 (42%)	12 (71%)
≥ 5	9 (31%)	6 (50%)	3 (18%)
Unknown	3 (10%)	1 (8%)	2 (12%)

Median number of EV cycles received was 5 (range: 1-28). Median follow-up from EV start in the overall cohort was 16.3 months, PFS from EV treatment start was 5.1 months (95% CI: 3.91 to 19.3) and OS was 10.2 months (95% CI:5.78 to Not Reached).

### Genomic factors

Genomic data were available for 27 of the 29 patients. Thirteen genes were found to be altered in 10% or more of the patients (**Table 2**). Most common alterations in this dataset involved *TERT* promoter (*TERTp* n=20), *CDKN2A*/*CDKN2B* (n=11), and *TP53* (n=8). Compared to non-responders, responders were enriched for alterations in *TP53* (58% vs 7%; p <0.01), *KDM6A* (42% vs 7%, p =0.03) and *MDM2* (25% vs 0%, p=0.04). Correspondingly, an increased ORR to EV treatment was observed in mutated tumors compared to wild-type (**Table 2**).

**Table 2:** Genomic Characteristics - Univariable analysis of observed response, progression-free survival, and overall survival.

Alteration		ORR	p-value	mOS: HR (95% CI)	p-value	mPFS: HR (95% CI)	p-value
*ARID1A* (n = 8)	Present	50%	0.71	1.39 (0.47-4.18)	0.55	1.21 (0.49-3.21)	0.63
	Absent	42%					
*CDKN2A* (n = 11)	Present	36%	0.48	1.42 (0.49-4.12)	0.52	1.78 (0.72-4.40)	0.22
	Absent	50%					
*CDKN2B* (n = 11)	Present	36%	0.48	1.42 (0.49-4.12)	0.52	1.78 (0.72-4.40)	0.22
	Absent	50%					
*ERBB2* (n = 5)	Present	60%	0.44	0.21 (0.03-1.62)	0.14	0.53 (0.15-1.81)	0.31
	Absent	41%					
*KDM6A* (n = 6)	Present	83%	**0.03**	0.68 (019-2.45)	0.56	0.85 (0.31-2.35)	0.75
	Absent	33%					
*KM2TD* (n = 3)	Present	67%	0.41	0.47 (0.06-3.63)	0.47	0.72 (0.16-3.13)	0.66
	Absent	42%					
*MDM2* (n = 3)	Present	100%	**0.04**	0.79 (0.18-3.61)	0.77	0.85 (0.25 -2.96)	0.80
	Absent	38%					
*MTAP* (n = 3)	Present	67%	0.95	0.52 (0.07-2.96)	0.52	2.56 (0.69-9.55)	0.16
	Absent	42%					
*PIK3CA* (n = 7)	Present	43%	0.92	0.73 (0.20-2.62)	0.63	0.94 (0.34-2.61)	0.90
	Absent	45%					
*RB1* (n = 4)	Present	75%	0.18	0.93 (0.21-4.22)	0.93	0.93 (0.27-3.23)	0.91
	Absent	39%					
*STAG2* (n = 3)	Present	67%	0.95	0.63 (0.08-4.84)	0.66	1.0 (0.23-4.38)	0.99
	Absent	42%					
*TERT* (n = 20)	Present	50%	0.33	0.52(0.16-1.72)	0.28	0.59 (0.21-1.67)	0.32
	Absent	29%					
*TP53* (n = 8)	Present	88%	**<0.01**	0.26 (0.06-1.18)	0.08	0.34 (0.11-1.02)	**0.05**
	Absent	26%					
*TP53/MDM2* (n=11)	Present	91%	**<0.01**	0.30 (0.09-0.99)	**0.05**	0.34 (0.13-0.90)	**0.03**
	Absent	13%					

n is the number of patients with the alterations in a specific gene.Bold values indicate that the statistical significance was met.

The univariable analysis showed alterations in *TP53* were associated with a longer PFS and trending towards a longer OS (**Table 2**). Furthermore, the multivariable analysis, while accounting for the relevant patient clinical characteristics, showed that *TP53* alteration was the only independent genomic marker predictive of improved PFS (**Table 3**).

**Table 3:** Multivariable analysis of overall survival and progression free- survival with relevant clinical variables and *TP53* alterations.

Characteristics at Baseline	mOS: HR (95% CI)	p-value	mPFS: HR (95% CI)	p-value
Age	0.85 (0.76 – 0.95)	**< 0.01**	0.94 (0.89 – 0.99)	**0.05**
Race (Non-White vs White)	0.71 (0.10 – 5.09)	0.74	0.24 (0.06 -1.02)	**0.05**
Histology (variant histology vs pure urothelial)	1.29 (0.25 – 6.6)	0.76	6.51 (1.60 – 26.50)	**<0.01**
ECOG PS (≥ 2 vs 0 or 1)	9.56 (1.77-51.46)	**<0.01**	9.23 (2.46 - 34.70)	**<0.01**
Visceral Mets (present vs absent)	1.11 (0.19 - 6.52)	0.90	7.96 (1.61 – 39.29)	**0.01**
Albumin (≥ 3g/dL vs < 3.0 g/dL)	0.09 (0.02 – 0.53)	**<0.01**	0.34 (0.09 – 1.27)	0.11
*TP53* alteration (present vs absent)	0.18 (0.02 – 1.90)	0.16	0.04 (0.004 – 0.39)	**<0.01**

Bold values indicate that the statistical significance was met.

**Figure 1:**
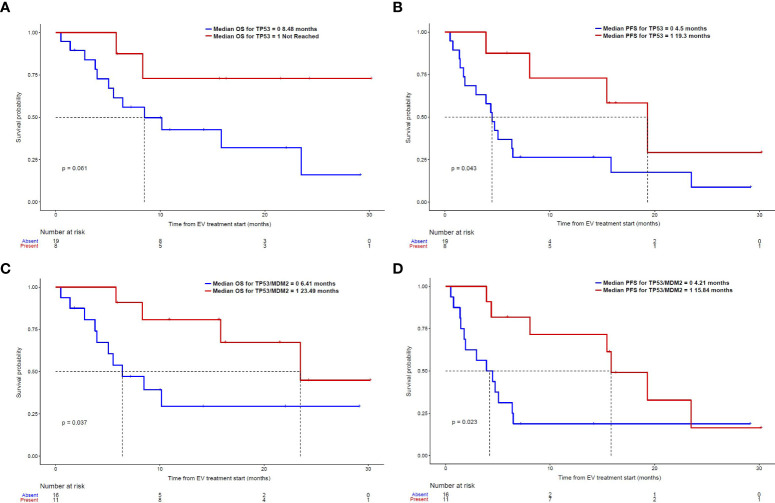
Kaplan-Meier curves: **(A)** OS and **(B)** PFS in patients with and without *TP53* alterations; **(C)** OS **(D)** PFS in patients with and without the composite *TP53/MDM2* alterations.

Given that *MDM2* alterations were found only in responders to EV and none of the non-responders in our dataset, and the fact that MDM2 is a well-established modulator of p53 (25), we further assessed the composite biomarker of alterations in either *TP53* or *MDM2* in both univariable and multivariable analyses. A total of 11 non-overlapping patients had this composite biomarker (8 with *TP53* alterations and 3 with *MDM2* alterations). This composite biomarker was associated with an increased ORR to EV treatment, longer PFS and OS on the univariable analysis (**Table 2**), as well as a longer PFS on the multivariable analyses (**Table 4**). **Figure 1** shows the Kaplan Meier curves for patients with and without *TP53* alterations and the composite *TP53/MDM2* alterations.

**Table 4:** Multivariable analysis of overall survival and progression free- survival with relevant clinical variables and composite *TP53/MDM2* biomarker.

Characteristics at Baseline	mOS: HR (95% CI)	p-value	mPFS: HR (95% CI)	p-value
Age	0.84 (0.75-0.94)	**<0.01**	0.95 (0.89-1.02)	0.15
Race (Non-White vs White)	1.41 (0.23-8.52)	0.71	0.92 (0.28-3.01)	0.90
Histology (variant histology vs pure urothelial)	0.83 (0.17-3.96)	0.81	2.93 (0.94-9.13)	0.06
ECOG PS (≥ 2 vs 0 or 1)	7.46 (1.54-36.15)	**0.01**	6.03 (1.78-20.43)	**<0.01**
Visceral Mets (present vs absent)	0.67 (0.13-3.42)	0.63	3.98 (0.97-16.30)	**0.05**
Albumin (≥ 3g/dL vs < 3.0 g/dL)	0.09 (0.014-0.63)	**0.01**	0.70 (0.17-2.92)	0.62
*TP53/MDM2* biomarker (present vs absent)	0.49 (0.09-2.55)	0.39	0.19 (0.04-0.92)	**0.04**

Bold values indicate that the statistical significance was met.

**Figure 2:**
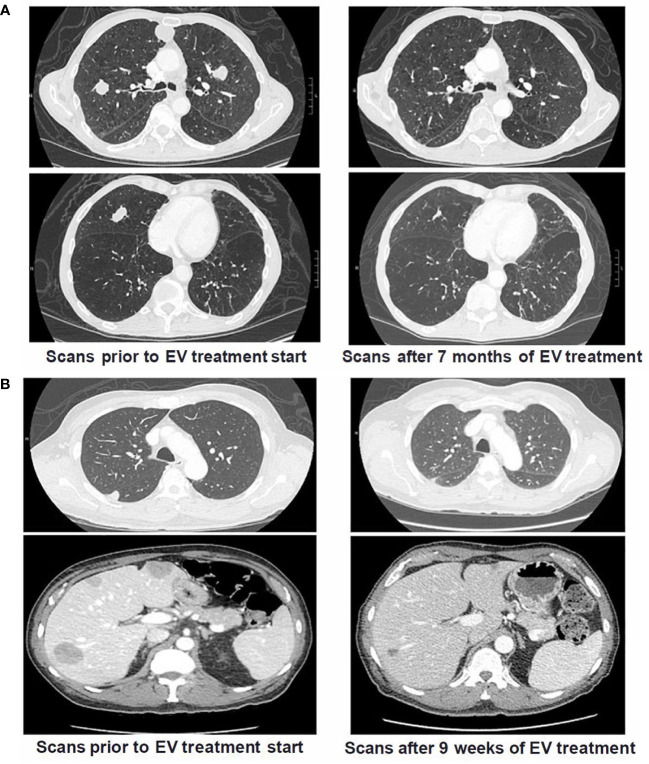
Cross Sectional Imaging of Patients with *TP53* and *MDM2* Somatic Alterations Pre and Post Treatment with Enfortumab Vedotin. **(A)** Patient with metastatic UC and *TP53* alterations (p.E285* and p.E271Q) who had prior progression on both platinum-based chemotherapy and pembrolizumab. Patient had significant decrease in size of biopsy-confirmed pulmonary metastases after starting treatment with EV and eventually achieved a complete response. Patient continues to have radiographic CR on scans 30 months after EV treatment start. **(B)** Patient with metastatic UC and *MDM2* alteration (amplification) who had prior progression on a clinical trial of an immune checkpoint inhibitor combination regimen with metastases in lungs and liver. Following start of EV treatment, patient had marked reduction of metastatic burden after only 9 weeks and continued on treatment for >18 months.

Several patients with *TP53* or *MDM2* alterations had dramatic responses to treatment with EV. Radiographic responses of a patient with a *TP53* alteration and another patient with *MDM2* alteration treated with EV are illustrated on **Figures 2A, B**, respectively.

### Clinical factors

On the univariable analysis (**Table 5**), albumin level ≥ 3.0g/dL was associated with a prolonged OS while ECOG PS < 2 at baseline was associated with a prolonged PFS.

**Table 5:** Clinical Characteristics - Univariable analysis of progression-free survival and overall survival.

Characteristics at Baseline	mOS: HR (95% CI)	p-value	mPFS: HR (95% CI)	p-value
Age	0.94 (0.88 – 1.0)	0.06	0.96 (0.91 -1.02)	0.17
Race (Non-White vs White)	1.6 (0.59 – 4.37)	0.34	2.13 (0.89 -5.03)	0.08
Histology (variant histology vs pure urothelial)	0.77 (0.28-2.08)	0.60	1.49 (0.63 – 3.55)	0.36
Primary Tumor location (Other vs Bladder)	0.97 (0.31 -3.05)	0.96	0.83 (0.30 – 2.26)	0.70
ECOG PS (≥ 2 vs <2)	1.9 (0.69-5.33)	0.21	2.64 (1.1-6.37)	**0.03**
Visceral Mets (Yes vs No)	1.6 (0.51-4.93)	0.43	2.13 (0.78 – 5.82)	0.14
Smoker Status (former/current vs never)	0.77 (0.28 – 2.15)	0.62	0.75 (0.31 – 1.78)	0.51
BMI ≥ median (24.05 kg/m²)	1.8 (0.64 - 5.1)	0.26	1.27 (0.53 – 3.02)	0.59
Albumin ≥ 3.0 g/dL	0.28 (0.08-0.97)	**0.04**	0.69 (0.27-1.82)	0.46
NLR ≥5	1.98 (0.55-7.07)	0.29	1.26 (0.49-3.73)	0.56
Hemoglobin ≥ 10 g/dL	0.64(0.21-1.91)	0.42	0.91 (033-2.53)	0.86
eGFR ≥60	0.47(0.13-1.67)	0.24	0.74 (0.29-1.93)	0.54
Bellmunt Criteria ≥ 2	2.3 (0.8-6.4)	0.12	1.86 (0.78-4.41)	0.16
Frontline ICI risk score ≥ 2	2.7 (0.77-9.66)	0.12	12.49 (0.91-6.87)	0.07

Bold values indicate that the statistical significance was met.

In both of the multivariable analysis models, which included *TP53 and TP53/MDM2* composite alterations respectively (**Tables 3**, **4**), older age (continuous variable), better ECOG PS scores (< 2), and higher albumin levels (≥3 g/dL) were found to be independent predictors of a prolonged OS. In both models, better ECOG PS scores (<2) and absence of visceral metastases were also independently associated with prolonged PFS. In the multivariable model including *TP53* alterations (**Table 3**) only, additional factors predictive of prolonged PFS included non-white race, pure urothelial histology, and older age.

## Discussion

In this single-center retrospective analysis of 29 patients with aUC treated with enfortumab vedotin, somatic alterations in *TP53* and *MDM2* were independently associated with improved outcomes with EV treatment, while controlling for the relevant clinical and laboratory variables. This intriguing observation in a treatment setting where predictive biomarker data have been lacking, deserves further validation in independent patient cohorts. Despite the relatively smaller sample size of this cohort, the observed response rate of 41%, as well as mPFS of 5.1 months, and mOS of 10.2 months are consistent with data previously reported by larger clinical trials and retrospective studies in this patient population (9, 12, 26). Consequently, this data, while hypothesis-generating, sheds important light on the biomarker landscape in aUC, where treatment decisions must frequently be made in the refractory setting among the several therapeutic options available.

EV is thought to have anti-tumor activity by targeting cells that express Nectin-4. A preclinical study suggests tumor Nectin-4 expression is associated with increased EV activity, nominating Nectin-4 as a potential biomarker of response to EV (27). However, similar data has not been presented from clinical trials of patients treated with EV. A recent publication reported decreased PFS with EV treatment in patients with absent or weak membranous Nectin-4 expression, providing the first data for Nectin-4 expression as a predictive biomarker (28). There is limited evidence of other biomarkers of response to EV, and the role of genomic alterations as predictive biomarkers in this space has not been well studied.

Data from our patient cohort suggests *TP53* as a potential biomarker of improved outcomes with EV treatment. *TP53* encodes for p53, a tumor suppressor that plays a profound role in cancer. *TP53* is found to be one of the most commonly altered genes in UC and is associated with poor disease outcomes (29–32). Interestingly, in our study we noted improved outcomes in patients with *TP53* alteration to EV treatment. We observed an association between *TP53* alterations and longer OS and PFS. Presence of *TP53* alterations remained significantly associated with PFS in multivariable analysis after adjusting for clinical and laboratory characteristics. *TP53* mutations in UC are known to be highly variable. Among patients with *TP53* alterations (n=8) in our cohort, patients had either 1 (n=4) or 2 (n=4) alterations in the *TP53* gene, with no two patients having the same alteration. The types of mutations present were either a point mutation (missense, stop gain, or splice site mutation), a deep deletion, or both. Responders (n=7) had the following *TP53* alterations: p. G334fs; p.R175H; p.E285* and p.E271Q; p.R248W; p.S166* and p.R273C; p.E271K; p.S149F and a deep deletion, while the one non-responder had p.A276G and splice site c.673-2A>G. With the caveat of a small sample size, it appears that benefit with EV treatment can occur regardless of the type of *TP53* alteration present.

In addition to *TP53*, we noted *MDM2* alterations to be enriched in EV responders. *MDM2* is a negative regulator of p53 and promotes the rapid degradation of p53 (33–35). In our dataset, all 3 patients with *MDM2* alterations had amplifications, consistent with amplification being the most frequent alteration type involving *MDM2* in bladder cancer, which leads to a reduction or loss of function of p53 (36, 37). Furthermore, *MDM2* alterations were found to be mutually exclusive to *TP53* alterations in our dataset, and with the same direction of effect, thus strengthening the association between the composite *TP53/MDM2* alteration biomarker and EV response. Our findings, if validated in additional independent patient cohorts, raise a plausible hypothesis that urothelial cancers with a reduced or loss of function of the MDM2-p53 pathway may respond particularly well to EV.

The other common alterations, in addition to *TP53*, present in our dataset included *TERT* promoter*, CDKN2A/CDKN2B*, and *KDM6A*, consistent with what has previously been reported. For patients with UC, the presence of *TERT* promoter alterations is associated with improved outcomes to ICI treatment (38), although another recent study did not replicate these results (39). On the other hand, *CDKN2A/B* alterations have been associated with increased resistance to ICI and chemotherapy (40–42). Neither *TERT* promoter nor *CDKN2A/CDKN2B* alterations were found to be associated with EV treatment outcomes in our analysis. However, KDM6A alterations were found to be enriched in responders, although no association with survival outcomes was noted.

In terms of clinical factors, our findings suggest that higher albumin levels (≥ 3.0 g/dL) and better ECOG performance score (0-1 vs ≥ 2) were associated with improved outcomes on both univariable and multivariable analyses. These findings are concordant with previously reported data with EV. In a previous analysis of 49 patients with mUC treated with EV, lower albumin levels at 4-7 weeks from therapy initiation and ECOG PS 1 (vs 0) at treatment start was associated with worse OS (43). However, high albumin levels and ECOG status may be biomarkers predictive of improved response in UC regardless of the treatment a patient may receive. Previous analyses have found low albumin levels predict inferior outcomes to ICI in patients with mUC and better ECOG performance scores (<2) predict improved outcomes to ICI in patients with aUC (38, 44). Another analysis of EV outcomes in a multicenter cohort UNITE study reported consistent responses to EV in patients with both ECOG PS < 2 and ≥ 2 respectively (26). The same UNITE study dataset noted numerically higher responses to EV in patients with a pure urothelial histology as compared to patients with a variant histology component (58% vs 42%, p=0.06). These findings are consistent with the results presented in our current analysis, as we found pure UC histology to be associated with prolonged PFS in a multivariate analysis.

Interestingly our findings also suggested that older patients may have improved outcomes with EV treatment. Older patients are generally thought to have inferior treatment outcomes regardless of therapy, although in the UNITE dataset no differences in response to EV were seen based on age (26). As this was a dataset of mostly significantly pretreated patients, one potential explanation for these findings is that older patients included in the current analysis may have had a less aggressive disease biology leading them to still qualify for EV monotherapy even after having received multiple prior lines of treatment. Such patients would potentially then be more likely to have better outcomes with EV treatment as well. These findings do suggest that there should not be an age cutoff or an exclusion criterion for treatment with EV.

Other potential characteristics associated with treatment outcomes in patients with mUC include burden of metastatic disease and metastatic sites. In the UNITE study, presence of liver metastases was associated with an increased response rate to EV, but a shorter mOS (26). In our analysis, the number of patients with liver metastases were too small to assess this as an independent marker, however liver metastases were included within the criteria we did assess, including presence of visceral metastases, Bellmunt criteria and frontline ICI score (23, 24). The findings from both of our multivariate models including *TP53* and *TP53/MDM2* respectively, indicated visceral metastases to be associated with inferior outcomes, specifically a shorter PFS.

The main limitations are the study’s retrospective nature and the relatively small sample size of our cohort. This study is limited to a single academic institution which makes it more challenging to generalize the findings to other academic and community sites. While assessing the radiographic response, there was no central blinded review. Tumor mutational profiling was completed using two different NGS platforms, and as a result, identical gene panels were not utilized to assess for alterations in patients, which will also introduce some heterogeneity in results.

Despite these limitations, our findings nominate *TP53* and *MDM2* alterations as potential novel biomarkers of response to EV for patients with aUC. These findings, if validated, may importantly facilitate patient selection for EV treatment since *TP53* alteration is known to be a negative prognostic biomarker. This analysis also lends further support to using baseline albumin level and ECOG PS as predictive markers of response to EV therapy, in addition to their established role in prognostication. These findings are hypothesis generating, and both external and prospective validation of these results are needed in larger patient cohorts. However, these initial findings in a treatment space currently mostly devoid of biomarkers may still potentially inform future studies and clinical decision making for patients with aUC.

## Data availability statement

The raw data supporting the conclusions of this article will be made available by the authors, without undue reservation.

## Ethics statement

The studies involving human participants were reviewed and approved by UCSF IRB. Written informed consent for participation was not required for this study in accordance with the national legislation and the institutional requirements.

## Author contributions

Conceptualization: VSK, and TJ. Methodology: VSK and TJ. Data Curation: TJ, IK, PD, CS, VK, XZ, EM, SF, MJ, AA, DK, AD, HB, RB, AW, SP, RA, ES, LF, JC, TF and VSK Formal Analysis: TJ. Writing – Original Draft Preparation: TJ and VSK. Writing – Review & Editing: TJ, IK, PD, CS, VK, XZ, EM, SF, MJ, AA, DK, AD, HB, RB, AW, SP, RA, ES, LF, JC, TF and VSK. All authors contributed to the article and approved the submitted version.
